# Nanovesicles from *Malassezia sympodialis* and Host Exosomes Induce Cytokine Responses – Novel Mechanisms for Host-Microbe Interactions in Atopic Eczema

**DOI:** 10.1371/journal.pone.0021480

**Published:** 2011-07-22

**Authors:** Ulf Gehrmann, Khaleda Rahman Qazi, Catharina Johansson, Kjell Hultenby, Maria Karlsson, Lena Lundeberg, Susanne Gabrielsson, Annika Scheynius

**Affiliations:** 1 Clinical Allergy Research Unit, Department of Medicine Solna, Karolinska Institutet, Stockholm, Sweden; 2 Department of Laboratory Medicine, Karolinska University Hospital Huddinge, Huddinge, Sweden; 3 Dermatology and Venereology Unit, Karolinska University Hospital, Stockholm, Sweden; Centre de Recherche Public de la Santé (CRP-Santé), Luxembourg

## Abstract

**Background:**

Intercellular communication can occur via the release of membrane vesicles. Exosomes are nanovesicles released from the endosomal compartment of cells. Depending on their cell of origin and their cargo they can exert different immunoregulatory functions. Recently, fungi were found to produce extracellular vesicles that can influence host-microbe interactions. The yeast *Malassezia sympodialis* which belongs to our normal cutaneous microbial flora elicits specific IgE- and T-cell reactivity in approximately 50% of adult patients with atopic eczema (AE). Whether exosomes or other vesicles contribute to the inflammation has not yet been investigated.

**Objective:**

To investigate if *M. sympodialis* can release nanovesicles and whether they or endogenous exosomes can activate PBMC from AE patients sensitized to *M. sympodialis*.

**Methods:**

Extracellular nanovesicles isolated from *M. sympodialis*, co-cultures of *M. sympodialis* and dendritic cells, and from plasma of patients with AE and healthy controls (HC) were characterised using flow cytometry, sucrose gradient centrifugation, Western blot and electron microscopy. Their ability to stimulate IL-4 and TNF-alpha responses in autologous CD14, CD34 depleted PBMC was determined using ELISPOT and ELISA, respectively.

**Results:**

We show for the first time that *M. sympodialis* releases extracellular vesicles carrying allergen. These vesicles can induce IL-4 and TNF-α responses with a significantly higher IL-4 production in patients compared to HC. Exosomes from dendritic cell and *M. sympodialis* co-cultures induced IL-4 and TNF-α responses in autologous CD14, CD34 depleted PBMC of AE patients and HC while plasma exosomes induced TNF-α but not IL-4 in undepleted PBMC.

**Conclusions:**

Extracellular vesicles from *M. sympodialis*, dendritic cells and plasma can contribute to cytokine responses in CD14, CD34 depleted and undepleted PBMC of AE patients and HC. These novel observations have implications for understanding host-microbe interactions in the pathogenesis of AE.

## Introduction

Exosomes are nanovesicles (30–100 nm) of endosomal origin produced by different cells [1], [2], [3]. They can be secreted into the extracellular space and act as means of intercellular communication by transferring functional proteins and RNA molecules between cells [4], [5]. They can also carry antigens from microorganisms such as viruses and bacteria [6], [7]. Exosomes can be isolated from body fluids such as plasma [8], bronchoalveolar lavage (BAL) fluid [9], breast milk [10] and urine [11]. Previous studies have found that B-cells and dendritic cells (DC) release MHC class II containing exosomes which could activate immune responses *in vitro* and *in vivo* [12], [13]. Due to their immunostimulatory capacity, exosomes from antigen presenting dendritic cells (APC) are currently being tested as vaccine vehicles in cancer [14], [15], [16], [17] and against infections [18], [19]. However, it is still unclear which role exosomes have *in vivo* and during human inflammatory diseases. Only one previous study has investigated the immunostimulatory effects of exosomes from cells of patients with allergy. Here, B-cell exosomes directly loaded with birch pollen allergen could induce Th2-cytokine responses in PBMC of sensitised patients [20].

Atopic eczema (AE) is a common chronic inflammatory skin disease. While the pathogenesis of the disease remains unclear, studies suggest that a genetic predisposition in combination with defects in the skin barrier facilitate the development of AE [21], [22]. A defective skin barrier in turn might assist the entry of microorganisms which can trigger symptoms by acting as allergens. One such microorganism is the lipophilic yeast *M. sympodialis*, which belongs to our common cutaneous microbial flora [23]. 30–80% of adult AE patients are reactive to *M. sympodialis* in terms of specific IgE- and T-cell reactivity and/or positive atopy patch test (APT) reactions indicating a link between AE and *M. sympodialis* [24]. Sensitization to *M. sympodialis* is most likely mediated by APC such as DC in the skin. We have previously found that human monocyte-derived dendritic cells (MDDC) rapidly internalize *M. sympodialis* [25] and in response upregulate the maturation marker CD83 and the co-stimulatory molecules CD80 and CD86 [26]. Furthermore *M. sympodialis* induces lymphocyte proliferation and a Th2-like immune response in sensitized AE patients [27].

Recently, the release of extracellular vesicles has been described for the fungi *Cryptococcus neoformans*, *Histoplasma capsulatum* and *Saccharomyces cerevisiae* [28], [29]. It is speculated that these vesicles function in enzymatic nutrient degradation and in host-microbe interactions [30], e.g. by modulating murine macrophage functions *in vitro* [31].

In this study we hypothesised that *M. sympodialis* releases nanovesicles with immune modulating functions. We compared their capacity to induce cytokine responses in autologous PBMC of AE patients and healthy controls (HC) with that of exosomes derived from MDDC co-cultured with *M. sympodialis* or isolated from plasma. We demonstrate for the first time that *M. sympodialis* releases extracellular vesicles (MalaEx) carrying allergen. These vesicles induced IL-4 and TNF-α responses in PBMC with a significantly higher IL-4 production in the patients compared to the controls. Furthermore, we report that exosomes from MDDC co-cultured with *M. sympodialis* (DCexo Mala) elicit IL-4 and TNF-α responses whereas plasma exosomes induced TNF-α but not IL-4 production in AE patients and HC. These novel findings suggest that nanovesicles, autologous or derived from fungi, serve diverse immunoregulatory functions which might contribute to the inflammation in AE.

## Methods

### Ethics Statement

The study was approved by the Regional Ethical Review Board in Stockholm and all participants gave their written informed consent.

### AE patients and healthy controls

Male AE patients and HC (Table 1) were recruited from the Stockholm area using the same inclusion and exclusion criteria as described previously [32] (see Online Repository S1). Patients and controls were asked to come back for a full blood donation of 450 ml for generation of MDDC, storage of plasma at −80°C and of PBMC at −150°C. The blood donations and following experiments were performed pairwise with one AE patient and one HC. Twelve additional healthy blood donors were included from the Karolinska University Hospital Solna Blood Bank.

**Table 1:** Characterisation of study subjects.

Individual	Age(years)	SubjectiveSCORAD1)	Plasma IgE(kU/L)2)	Phadiatop®,3)	*M.sympodialis* specificIgE (kU/L)4)
HC*1	25	-	22	Neg	<0.35
HC 2	57	-	6.5	Neg	<0.35
HC 3	63	-	8.7	Neg	<0.35
HC 4	25	-	11	Neg	<0.35
HC 5	22	-	14	Neg	<0.35
HC 6	43	-	6.9	Neg	<0.35
HC 7	41	-	8.5	Neg	<0.35
HC 8	24	-	35	Neg	<0.35
**Median (range)**	**33 (22–63)**		**9.9 (6.5–35)**		**<0.35**
AE†1	25	65	140	Pos	0.83
AE 2	20	68	1600	Pos	25
AE 3	39	76	11000	Pos	27
AE 4	57	42	1400	Pos	21
AE 5	51	48	2200	Pos	29
AE 6	31	73	680	Pos	11
AE 7	21	62	120	Pos	1.6
AE 8	33	51	36	Pos	2.3
**Median (range)**	**32 (20–57)**	**63.5 (42–76)**	**1040 (36–11000)**		**16 (1.6–29)**

* HC = Healthy control.

† AE = Atopic eczema patient.

1) SCORAD  =  Severity scoring of atopic eczema [48].

2) ImmunoCAP™ (Phadia AB), reference range 1.6–122 kU/L.

3) A positive Phadiatop® (Phadia AB) was defined as having plasma IgE to any of 11 common aeroallergens ≥0.35 kU/L.

4) Specific plasma IgE to *M. sympodialis* analysed with ImmunoCAP™ (m70 Phadia AB).

All values (except SCORAD) were determined at the time of blood donation (AE 3 and 4 donated blood twice; values of the first donation are displayed).

### Generation of monocyte derived dendritic cells (MDDC)

MDDC were generated as previously described [33] with some modifications. PBMC were depleted of CD34^+^ cells and CD14^+^ monocytes by magnetic bead separation (Miltenyi Biotech, Bergisch Gladbach, Germany) and the remaining cells were frozen (CD14, CD34 depleted PBMC). CD14^+^ monocytes were cultured for 6 days in exosome-free medium [34] supplied with 1 ng/ml IL-4 (Biosource International, Camarillo, CA) and 10 ng/ml GM-CSF (Invitrogen/GIBCO, Paisley, UK) for differentiation into MDDC. On day 6, cells were phenotyped by flow cytometry (see below). The cells showed a typical phenotype of immature MDDC as previously described [35] without any significant differences between AE patients and HC (see Online Repository S1).

### Culture of *M. sympodialis*

*M. sympodialis* (ATCC strain 42132) was cultured on modified Dixon agar plates [26] for 4 days at 32°C and contamination was excluded using blood and Sab-oxoid agar plates. Colonies were harvested and resuspended in RPMI-1640 (Hyclone, Logan, UT). The cells were washed once and counted by the trypan blue exclusion method. Supernatants from *M. sympodialis* cultured at a concentration of 2×10^6^ cells/ml for 48 hr at 37°C in exosome-free medium [34] were stored at −80°C for later nanovesicle preparation.

### Co-culture of MDDC with *M. sympodialis*

Immature MDDC were cultured with or without live *M. sympodialis*. After 48 hr, culture supernatants were harvested and kept at −80°C until exosome preparation and cells were phenotyped using flow cytometry (see below and Online Repository S1).

### Flow cytometric analysis of cells

MDDC were phenotyped before and after culture with *M. sympodialis* using flow cytometry and fluorescein-isothiocyanate (FITC)- or phycoerythrin (PE)-labelled mouse monoclonal antibodies (mAbs) using a FACS Calibur or FACS Aria (BDBiosciences, Bedford, MA) (see Online Repository S1).

### Nanovesicle preparations

Nanovesicles from *M. sympodialis* culture supernatants, the different MDDC culture supernatants and from plasma were prepared by serial ultracentrifugation as described previously [8], [35] with slight modifications (see Online Repository S1). Pellets were resuspended in PBS or RPMI, and protein content was measured using a Bradford assay (BioRad, Hercules, CA) (Table S1). Nanovesicle preparations were stored at −80°C.

### Characterisation of nanovesicles

Exosomes were phenotyped on anti-MHC class II coated Dynabeads (Invitrogen/Dynal, Paisley, UK) using mAbs and a FACS Calibur (BDBiosciences) [20]. To verify the presence of phenotypic cell markers or *M. sympodialis* antigens on vesicles, sucrose gradient analysis was performed as previously described [10] using uncoated latex beads (Invitrogen) or anti-MHC class II Dynabeads (Invitrogen/Dynal) (see Online Repository S1).

### Negative staining (TEM)

Three µL drops of DCexo, DCexo Mala or MalaEx samples were added to carbon coated formvar grids for 10 min. The excess solution was removed by a filter paper and grids were directly stained by 2% uranyl acetate for 10 sec followed by a quick rinsing in distilled water. Grids were analyzed in a Tecnai 12 transmission electron microscope (FEI company, Eindhoven, The Netherlands) and images were taken by a Veleta digital camera (Olympus Soft Imaging Solutions, GmbH, Münster, Germany).

### Immuno-negative staining (iEM)

DCexo, DCexo Mala or MalaEx coated grids were blocked from unspecific binding by floating grids face down on drops of 10% normal goat serum (NGS) in 0.1 M phosphate buffer (PB) for 10 min followed by incubation with a mAb against HLA-DR (BD Biosciences) or a purified rabbit anti-*M. sympodialis* Ab (generated in house) for 2 hr. As controls, the primary antibodies were replaced by IgG_2a_ (BDBiosciences) and normal rabbit IgG from non-immunized mice (DAKO Cytomation, Glostrup, Denmark). Grids were washed several times in phosphate buffer containing 0.1% NGS and bound antibodies were detected by secondary gold-particle conjugated goat anti-rabbit (10 nm) and anti-mouse (5 nm) (Biocell, BBInternational, Cardiff, England). Grids were stained and analyzed as described above.

### ELISPOT and ELISA analyses

CD14, CD34 depleted PBMC were cultured alone, or with nanovesicles from *M. sympodialis*, or with exosomes from MDDC cultured alone, or co-cultured with *M. sympodialis* at different concentrations while PBMC were cultured with plasma exosomes in the absence or presence of *M. sympodialis*. IL-4 ELISPOT assays were performed in triplicates for 48 hr according to the manufacturer's instructions (Mabtech, Stockholm, Sweden) and analysed by an ELISPOT automated reader (AID Diagnostika, Straßberg, Germany) (see Online Repository S1). ELISPOT supernatants were harvested and analysed for TNF-α using ELISA according to the manufacturer's instructions (Mabtech). The detection limit was 8 pg/ml. Results are expressed as the mean of triplicates.

### Western Blot

The pelleted sucrose gradient fractions from *M. sympodialis* extracellular vesicles were denatured in Laemmli sample buffer (BioRad), separated on SDS polyacrylamide gels (BioRad) and the separated proteins were transferred to polyvinylidene difluoride membrane (Millipore) using a semi dry blotting system (BioRad). *M. sympodialis* specific bands were detected by using polyclonal rabbit IgG raised against *M. sympodialis* extract (generated in house) in 1∶2000 dilution, or serum from a patient with AE, sensitized to *M. sympodialis* (*M. sympodialis* specific IgE (m70, Phadia AB) 39 kU/L, total serum IgE 1700 kU/L) in a 1∶5 dilution. Serum from HC no 6 (Table 1) was used as negative control. Antibodies used for detection were rabbit anti-human IgE (1∶2000, MIAB Antibodies, Uppsala, Sweden) and horseradish peroxidase (HRP)-linked goat anti-rabbit IgG (1∶2000, Cell Signaling, Danvers, MA). Membranes were developed using the enhanced chemiluminescence (ECL) advanced detection kit (GE Healthcare).

### Statistical analysis

Nonparametric tests were performed using Graphpad Prism version 5 for Windows (Graphpad Software Inc., San Diego, CA, USA, www.graphpad.com). Wilcoxon matched pairs test was used within one group, and unpaired Mann-Whitney test between groups. A p-value<0.05 was considered statistically significant.

## Results

### *M. sympodialis* secretes extracellular vesicles which elicit IL-4 and TNF-α responses

**Figure 1:**
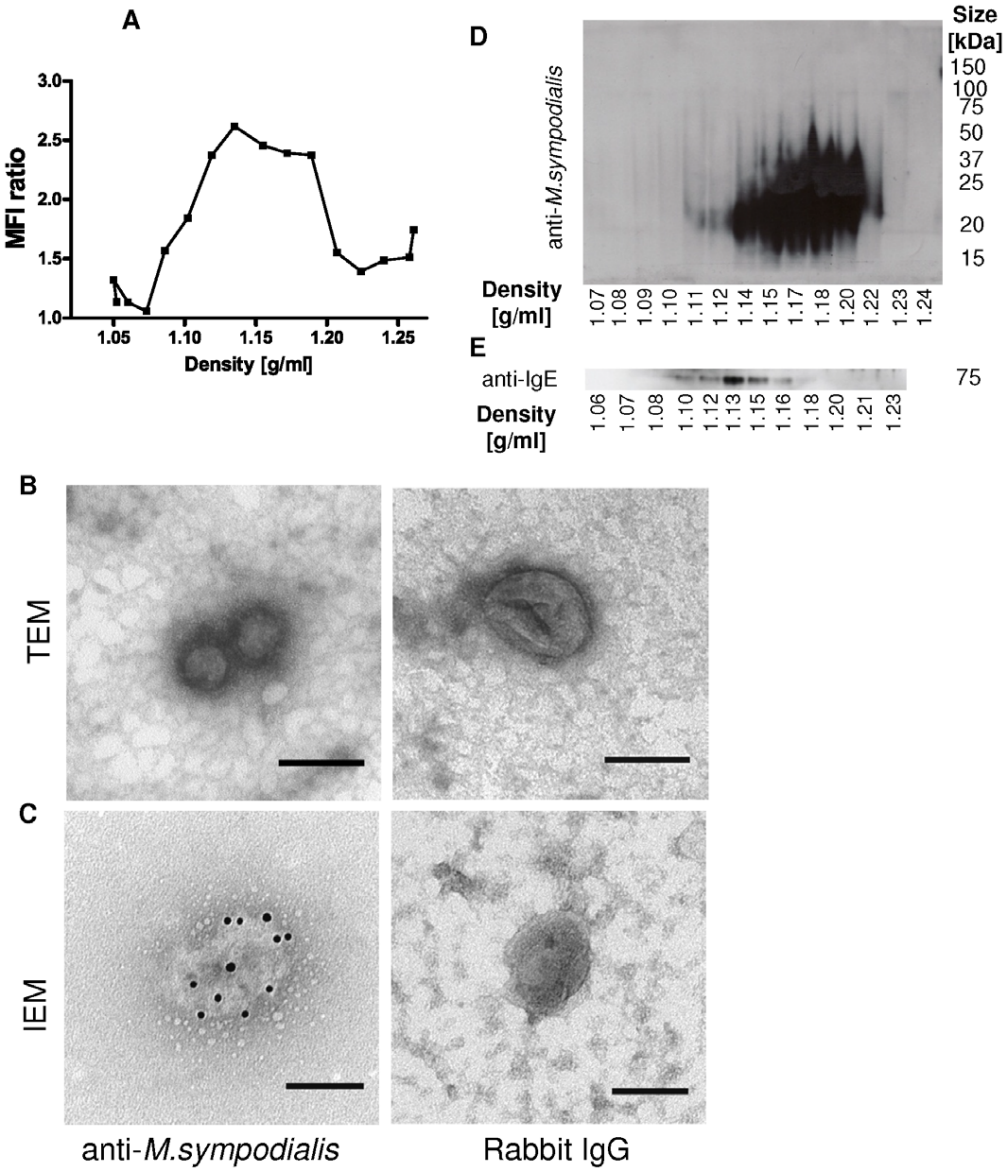
*M. sympodialis* releases extracellular vesicles. (**A**) *M. sympodialis* culture supernatants were ultracentrifuged and sucrose gradient fractions coated onto latex beads and analysed for the presence of *M. sympodialis* epitopes using flow cytometry. Data is from one representative out of 6 independent experiments and displayed as mean MFI ratio of a rabbit antibody against *M. sympodialis* and unspecific rabbit IgG. Fractions ranging in density from 1.11–1.20 g/ml were pooled and analysed by (**B**) TEM and (**C**) IEM with the same antibody as in A. Scale bars correspond to 100 nm. Pictures are representative in (**B**) of 4 independent experiments and in (**C**) of two independent experiments. (**D**) Western blot analysis of sucrose gradient fractions analysed using a rabbit antibody against *M. sympodialis* and (**E**) using serum from an AE patient sensitized to *M. sympodialis* to detect IgE-binding epitopes. Data shown in (D) and (E) are from one out of 2 experiments using separate sucrose gradients.

We first cultured *M. sympodialis* alone to test whether the yeast releases extracellular vesicles. Sucrose gradient fractions of pelleted culture supernatants revealed the presence of *M. sympodialis* derived antigens in density fractions ranging from 1.10 to 1.20 g/ml using flow cytometry (Fig. 1A). TEM analysis of these fractions showed the presence of vesicular structures with a size between 50–200 nm, with an average of approximately 100 nm (Fig. 1B). When performing iEM we could locate *M. sympodialis* antigens on these vesicular structures while controls showed negligible and unspecific labelling (Fig. 1C). Western blot analysis of the sucrose gradient fractions using polyclonal rabbit IgG raised against *M. sympodialis* showed the presence of antigens in vesicle fractions ranging in density from 1.12 g/ml to 1.22 g/ml (Fig. 1D) and 1.10–1.21 g/ml in two independent experiments, respectively. We then continued to look for IgE-binding epitopes using a separate sucrose gradient. When using serum from an AE patient containing specific IgE against *M. sympodialis* we could detect an IgE-binding protein with a size of approximately 70 kDa in fractions ranging in density between 1.10–1.18 g/ml (Fig. 1E) in two independent experiments. No bands were detected when using serum of HC no 6 (Table 1, data not shown). Thus, *M. sympodialis* can produce nanovesicles which contain antigens and among those also allergen.

**Figure 2:**
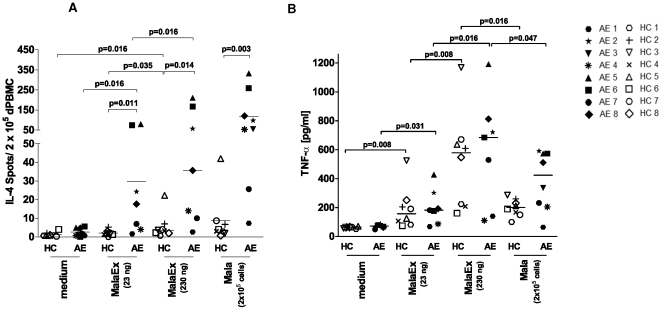
MalaEx elicit IL-4 and TNF-α responses. (**A**) IL-4 production in CD14, CD34 depleted PBMC as measured by ELISPOT from HC and AE patients when cultured alone (medium) or with MalaEx or *M. sympodialis* (Mala) for 48 hr. (**B**) TNF-α levels in the IL-4 ELISPOT supernatants were measured by ELISA. Data represent mean values of triplicates for each individual (Table 1); lines indicate median.

Next we assessed the effect of *M. sympodialis* vesicles, designated MalaEx, on CD14, CD34 depleted PBMC from AE patients and HC in an IL-4 ELISPOT assay for 48 hr. MalaEx significantly enhanced IL-4 production in CD14, CD34 depleted PBMC compared to the medium control in a dose dependent manner (Fig. 2A). The IL-4 responses were significantly higher in AE patients compared to HC both for MalaEx and for whole *M. sympodialis* cells (Mala) used as a control. MalaEx also induced a dose dependent TNF-α response in both AE patients and HC but without any significant differences between the two groups (Fig. 2B). These data indicate that *M. sympodialis* derived vesicles can elicit cytokine responses and that there is a difference in IL-4 reponse to MalaEx between HC and AE patients.

### Exosomes from MDDC and *M. sympodialis* co-cultures contain *M. sympodialis* antigens

Since DCs are among the first cells to encounter *M. sympodialis* in the skin, we prepared vesicles from MDDC and *M. sympodialis* co-cultures (DCexo Mala) and from MDDC alone (DCexo) to investigate the possible presence of *M. sympodialis* antigens on endogenous exosomes.

In agreement with a previous study, *M. sympodialis* induced maturation of MDDC, with a significant upregulation of CD80, CD83 and CD86 on MDDC [26], with no significant differences between AE patients and HC (Figure S1). *M. sympodialis* did not induce significant differences in the other molecules investigated (HLA-ABC, HLA-DR, CD11c, CD14, CD40, CD54 and CD63) compared to the medium control (data not shown).

**Figure 3:**
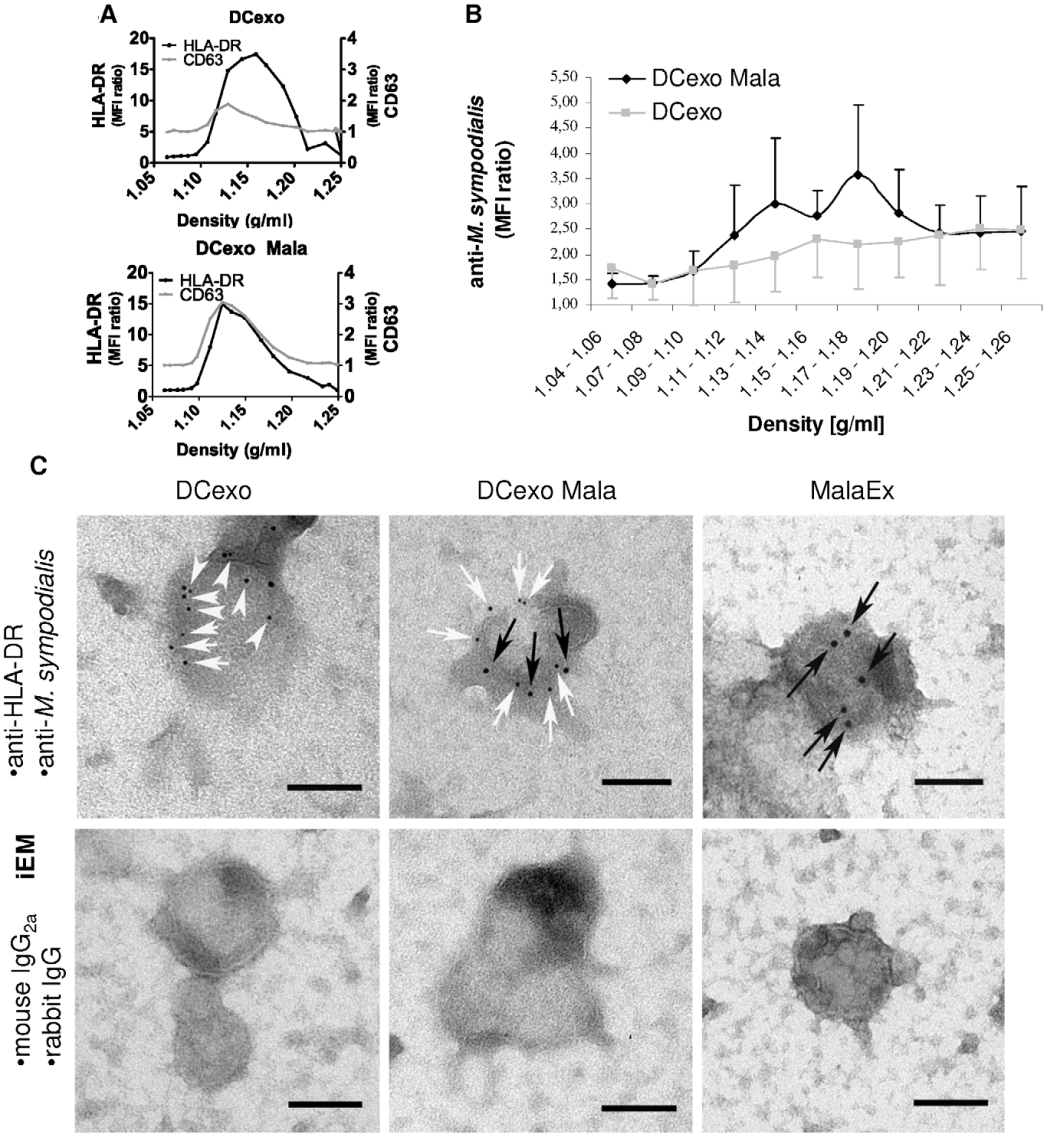
DCexo Mala contain *M. sympodialis* antigens. (**A**) Sucrose gradient fractions of exosomes from MDDC cultured alone (DCexo) or with *M. sympodialis* (DCexo Mala) were analysed for the expression of HLA-DR and CD63 using flow cytometry. Data are representative for one out of 3 experiments each using a pool of exosomes from 2 healthy blood donors and displayed as MFI ratio between specific antibody and isotype control. (**B**) Sucrose gradient fractions of DCexo and DCexo Mala analysed using flow cytometry. Data are from 5 independent experiments and displayed as mean MFI ratio ± SEM between a rabbit antibody against *M. sympodialis* and unspecific rabbit IgG. (**C**) Double IEM was performed on pools of DCexo and DCexo Mala from 5 AE patients and 3 HC as well as 2 independent preparations of MalaEx using a mAb against HLA-DR (detected with a 5 nm gold-particle conjugated goat anti-mouse Ab, white arrows) and a rabbit antibody against *M. sympodialis* (detected with a 10 nm gold-particle conjugated goat anti-rabbit Ab, black arrows) and the corresponding isotype controls. Scale bars correspond to 100 nm.

Flow cytometry analysis of sucrose gradient fractionated vesicles revealed the presence of HLA-DR and the tetraspanin CD63 in DCexo and DCexo Mala in fractions corresponding to exosomes (density 1.11–1.20 g/ml, [12], Fig. 3A). We could also detect *M. sympodialis* epitopes in sucrose gradient fractions of DCexo Mala ranging in density from 1.11–1.20 g/ml using flow cytometry, but not in the corresponding fractions of DCexo (Fig. 3B). Double iEM with a mAb to HLA-DR and a rabbit antibody to *M. sympodialis* revealed the presence of double positive vesicles in DCexo Mala preparations while the majority of the DCexo only bound the anti-HLA-DR antibody. Vesicles in MalaEx preparations only bound the antibody against *M. sympodialis* while isotype IgG controls showed negligible labelling (Fig. 3C).

### Exosomes from MDDC and *M. sympodialis* co-cultures induce IL-4 and TNF-α responses

**Figure 4:**
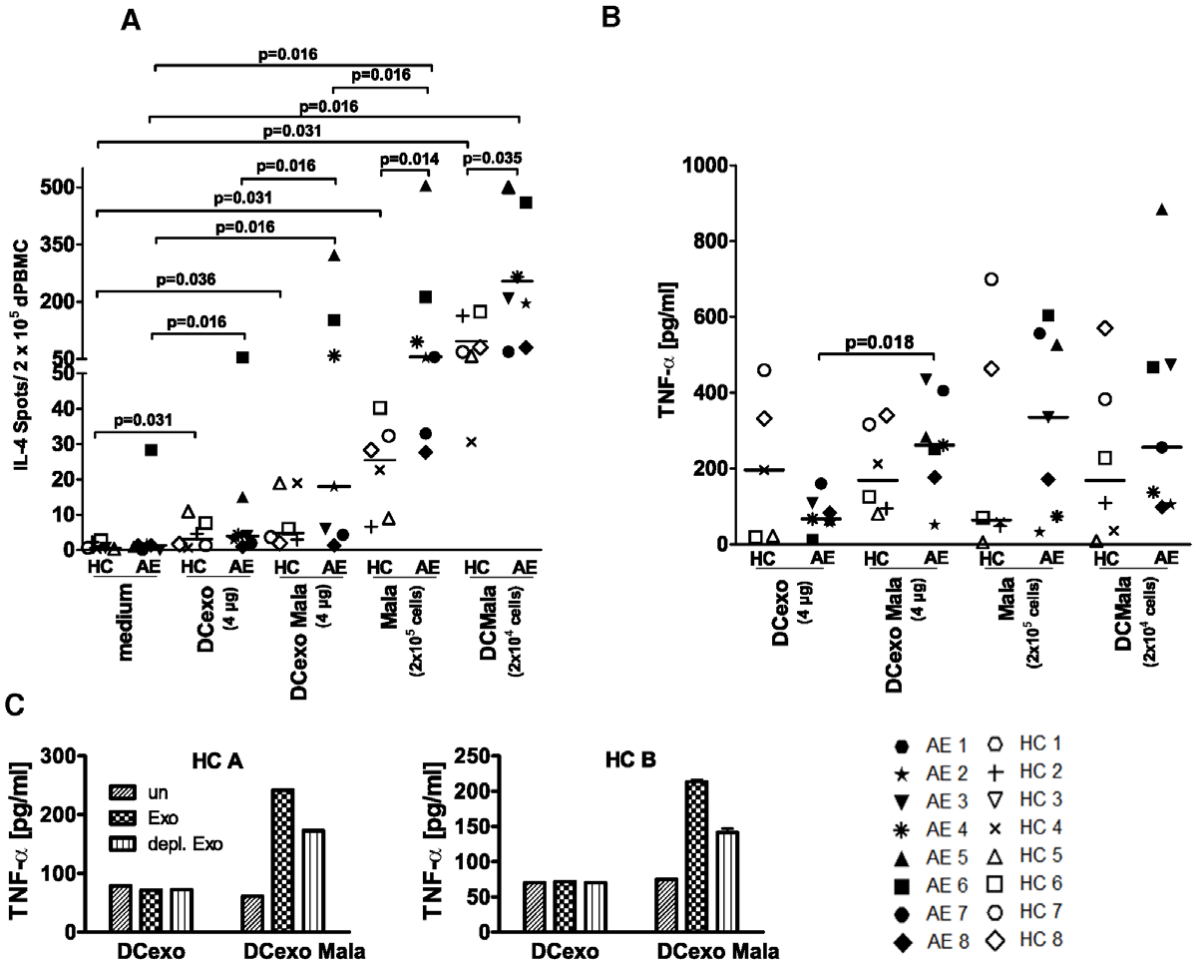
DCexo Mala elicit IL-4 and TNF-α responses. (**A**) IL-4 production in autologous CD14, CD34 depleted PBMC from AE patients and HC as measured by ELISPOT when cultured alone (medium), with DCexo, DCexo Mala, *M. sympodialis* (Mala) or MDDC co-cultured with *M. sympodialis* (DCMala) for 48 hr. (**B**) TNF-α levels in the IL-4 ELISPOT supernatants were measured by ELISA. Data represent mean values of triplicates for each individual (Table 1); lines indicate median. (**C**) TNF-α levels in supernatants of CD14, CD34 depleted PBMC of two additional healthy blood donors (HC A and HC B) when cultured alone (un), with DCexo or DCexo Mala (Exo) or with DCexo and DCexo Mala that were depleted of MHC class II positive vesicles (depl. Exo). Bars indicate mean value of duplicates.

To test whether endogenous nanovesicles have similar effects on cytokine responses as exogenous MalaEx, we added DCexo Mala in an IL-4 ELISPOT assay. We found that DCexo Mala induced significantly higher IL-4 responses than DCexo in autologous CD14, CD34 depleted PBMC from AE patients but not in CD14, CD34 depleted PBMC from HC (Fig. 4A). IL-4 responses were highest in response to *M. sympodialis* whole cells (Mala) and MDDC co-cultured with *M. sympodialis* (DCMala) with a statistically significant induction of IL-4 also in the HC group compared to the medium control (Fig. 4A). However, the levels were significantly lower than those observed in the AE patients. The TNF-α levels were significantly increased when stimulating CD14, CD34 depleted PBMC with DCexo Mala compared to DCexo in AE patients (Fig. 4B). A similar trend was seen for HC (Fig. 4B, C).

To investigate whether TNF-α induction indeed was due to DC-derived vesicles, exosome preparations from two additional healthy blood donors were depleted of MHC class II containing vesicles using anti-MHC class II Dynabeads. The DCexo Mala induced TNF-α levels were decreased by 38% (donor A) or 52% (donor B) after depletion (Fig. 4C), indicating that responses were at least in part due to DC-derived exosomes from the co-cultures.

### Plasma exosomes elicit TNF-α but not IL-4 in autologous PBMC

**Figure 5:**
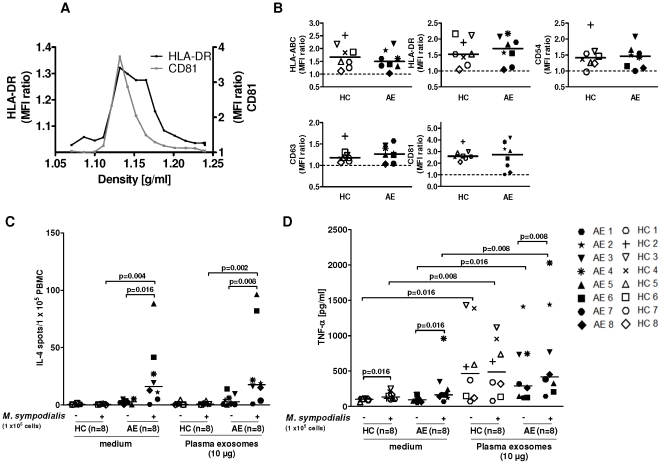
Plasma exosomes elicit TNF-α but not IL-4 responses. (**A**) Sucrose gradient fractions of plasma exosomes pooled from 2 healthy blood donors were loaded onto anti-MHC class II Dynabeads and analysed for the presence of HLA-DR and CD81 using flow cytometry. MFI ratio was calculated by dividing the sample MFI by the isotype control MFI. Data are from one out of two experiments. (**B**) plasma exosomes isolated from HC and AE patients were loaded onto anti-MHC class II Dynabeads and phenotyped using flow cytometry. Lines indicate the median. (**C**) PBMC were either cultured in only medium in the absence (−) or presence (+) of *M. sympodialis* or with autologous plasma exosomes in the absence (−) or presence (+) of *M. sympodialis*. IL-4 production in PBMC were measured by ELISPOT. (**D**) Culture supernatants from (C) were analysed for TNF-α levels using ELISA. Data represent mean values of triplicates for each individual (Table 1); lines indicate median.

Finally we prepared nanovesicles from plasma to find out whether their phenotype or function differed between AE patients and HC. Sucrose gradient analysis of plasma exosomes revealed the presence of CD81 and HLA-DR in density fractions ranging from 1.10 to 1.18 g/ml (Fig. 5A). Phenotypic analysis showed the presence of HLA-ABC, HLA-DR, CD54, CD63, and CD81 on plasma exosomes without any significant differences between AE patients and HC (Fig. 5B) while neither CD3, CD19, CD86, FasL, the B-cell activating factor of the TNF-family (BAFF) nor TGFβ1 could be detected on plasma exosomes (data not shown).

To assess whether these plasma exosomes could elicit cytokine responses they were added to autologous PBMC. In addition, we added *M. sympodialis* to the assay to see whether plasma exosomes could suppress or potentiate the immune response as seen in murine models of allergic sensitisation and delayed-type hypersensitivity [36], [37]. Plasma exosomes alone could neither elicit nor inhibit IL-4 responses in PBMC in any of the two groups at a concentration of 10 µg/ml (Fig. 5C). However, plasma exosomes as well as whole yeast cells significantly increased TNF-α production in PBMC of both AE patients and HC compared to the medium control and plasma exosomes enhanced the *M. sympodialis* induced TNF-α response in AE patients (Fig. 5D). These data suggest that plasma exosomes can contribute to innate immune responses in both AE patients and healthy controls.

## Discussion

Studies on the role of exosomes in human diseases are still hampered by the difficulties to isolate enough exosomes for functional studies in combination with their small size requiring demanding special techniques for their characterization [34]. We were here fortunate to obtain a full blood donation from the participants. Our study provides new insights into host-microbe interactions between a fungi and cells of our immune system. We found that exosomes from DCs co-cultured with *M. sympodialis* (DCexo Mala) carried *M. sympodialis* antigens and could stimulate cytokine production in autologous CD14, CD34 depleted PBMC from AE patients and HC. Furthermore, we show for the first time that *M. sympodialis*, a commensal yeast associated with AE [21], [24], secretes extracellular vesicles (MalaEx). These nanovesicles contain antigens and allergen from the fungi and can induce cytokine responses in CD14, CD34 depleted PBMC of AE patients and HC with a significantly higher IL-4 response in the *M. sympodialis* sensitized patients. The variation in IL-4 production between patients is in line with our previous study using extract of whole *M. sympodialis* cells [32]. Similar variation in cytokine responses in culture supernatants of PBMC stimulated with recombinant birch allergen, measured by cytometric bead array, has been reported for patients sensitised to birch pollen [38]. Our data suggest that extracellular vesicles, whether derived from *M. sympodialis* or from APC exposed to the fungi, may have a role in the pathogenesis of AE.

It has previously been shown that host exosomes can carry antigens from viruses and bacteria [6], [7] Exosomes from murine macrophages infected with intracellular pathogens have been found to contain pathogen associated molecular patterns (PAMPs) which could elicit pro-inflammatory responses *in vitro* and *in vivo* [6]. In case of infection with *Mycobacterium avium*, bacterial glycopeptidolipids could be traced through the endosomal compartment and were found in multivesicular bodies (MVBs) and on exosomes [39]. In analogy with this, *M. sympodialis* antigens might be processed by DCs and released on DC-derived exosomes which then induce cytokine responses.

With three different techniques we found *M. sympodialis* derived epitopes on MalaEx in vesicle containing sucrose gradient fractions ranging in density between 1.10–1.22 g/ml (Fig. 1A, D and E). By using patient serum we could detect an IgE-binding epitope with a size of approximately 70 kDa on MalaEx (Fig. 1E). Several IgE binding components in the 10–100 kDa molecular weight range have been identified in *Malassezia* and so far 10 *M. sympodialis* allergens have been cloned and sequenced (www.allergen.org, [24]). Thus, the observation that extracellular vesicles released by the yeast cells carry antigens and allergen and have the capacity to activate CD14, CD34 depleted PBMC raises questions on their role in the sensitization process and maintenance of the inflammatory response in AE. The nanovesicles are much smaller in size compared to whole yeast cells, which might lead to a different dissemination in the host and involve other mechanisms of cellular uptake and cell targeting. MalaEx were more potent than DCexo Mala to induce immune responses (cf Figs 2 and 4, and Table S2) which is why we speculate that MalaEx accumulate immunogenic molecules on their surface to enhance host-microbe interactions. Furthermore, a different set of molecules exposed on the surface of vesicles compared to that of yeast cells could lead to altered allergenicity. Interestingly, the elevated pH of AE skin [40] can induce an enhanced allergen release from *M. sympodialis* [41]. Future studies will show if altered pH will stimulate the production and allergenicity of *Malassezia* nanovesicles.

MalaEx and DCexo Mala also induced TNF-α responses in AE patients and HC. No statistically significant difference in TNF-α production were detected between AE patients and HC in response to any stimuli used (Table S2). It has previously been reported that *M. sympodialis* cells as well as the allergen Mala s 11 induce TNF-α production in MDDC and PBMC of healthy subjects [26], [42], [43]. This suggests that the TNF-α response is independent of previous sensitisation to *M. sympodialis* and represents an innate immune response. Our preliminary results indicate that TNF-α production in response to *M. sympodialis* or MalaEx involves NK-cells and possibly γδ-T-cells since an upregulation of the activation marker CD69 was seen on these cells (Gehrmann U. et al, unpublished observations).

It is reasonable to assume that exosomes from the host and extracellular vesicles from *M. sympodialis* coexist *in vivo* and contribute to host-microbe interactions. This might also be the case in our *in vitro* co-culture system. However, we believe that most of the immune responses recorded with DCexo Mala are induced by the exosomes from the DC and not the yeast for the following reasons: First, using iEM we found that the vast majority of vesicles in the DCexo Mala preparations were HLA-DR positive indicating their DC origin. Secondly, we have previously found that MDDC rapidly phagocytose live *M. sympodialis* cells within the first hr of culture [25] and we observed only few *M. sympodialis* cells in the medium after 48 hr of co-culture with MDDC using light microscopy (data not shown). Thirdly, by partially removing HLA-DR expressing DCexo Mala the TNF-α response was reduced by 40–50% (Fig. 3D). Due to limited amounts of exosomes we could not titrate the depletion with MHC class II beads but with a full titration an even stronger inhibition might have been seen.

Finally, we asked whether exosomes isolated from plasma could induce cytokine responses in PBMC. Human plasma exosomes are today mainly investigated as diagnostic markers for neoplastic diseases [44] and very little is known about the immunostimulatory or suppressive capacities of plasma exosomes. We found that plasma exosomes alone had no significant effect on IL-4 production indicating that they were not able to activate or suppress *M. sympodialis* specific responses. This might either be due to the absence of *M. sympodialis* antigens on plasma exosomes or to a small number of antigen-bearing plasma exosomes. Interestingly, plasma exosomes could induce TNF-α production in PBMC of both AE patients and HC (Fig. 5D) This induction could be due to the presence of PAMPs on exosomes, alternatively, pre-formed mRNA or mRNA stabilizing molecules for TNF-α [45]. One possible interpretation of these results is that plasma exosomes circulate as mediators capable of inducing a pro-inflammatory response when reaching high concentrations e.g. in local inflammatory settings. Since plasma exosomes contain leukotriene producing enzymes [46] the formation of pro-inflammatory lipid mediators and production of TNF-α might coincide and contribute to local inflammation. The capacity to induce TNF-α production has also been shown for other exosomes such as melanoma derived exosomes [47]. Although coming from different sources, the underlying mechanism for TNF-α induction might be similar for both types of exosomes.

In summary, we here present novel data that a microorganism releases extracellular vesicles that contain allergen and can contribute to inflammatory cytokine responses. This study also suggests that exosomes from DCs exposed to *M. sympodialis* can elicit IL-4 and TNF-α responses while plasma exosomes might be able to contribute to inflammation by inducing TNF-α. These observations have implications for understanding host-microbe interactions in the sensitisation and maintenance of the inflammation in AE.

## Supporting Information

Figure S1 *M. sympodialis* induces maturation in MDDC. Phenotypes of MDDC generated from AE patients and HC assessed by flow cytometry after 48 hr culture without (−) or with (+) *M. sympodialis* in a 1∶5 ratio. Data show median of mean fluorescence intensity (MFI) of the sample divided by the isotyope control, 25th–75th percentile and range.
(TIF)

Table S1 **Protein content^1)^ of vesicle preparations.** * HC = Healthy control. † AE = Atopic eczema patient. ^1)^as determined by Bradford assay (BioRad, Hercules, CA). ^2)^Exosomes were resuspended in RPMI. ^3)^Exosomes were resuspended in PBS.
(PPT)

Table S2 **Exosome-induced cytokine responses in AE patients and healthy controls.** § HC = Healthy control. † AE = Atopic eczema patient. ND = not done. ^1)^as determined by ELISPOT. ^2)^as determined by ELISA. *Mann-Whitney test to compare AE and HC, p-value<0.05.
(PPT)

Online Repository S1 (DOC)
