# Supplementary figures and images for: Nanovesicles from Malassezia sympodialis and Host Exosomes Induce Cytokine Responses – Novel Mechanisms for Host-Microbe Interactions in Atopic Eczema
Source: PLoS One. 2011 Jul 22;6(7):e21480. doi: 10.1371/journal.pone.0021480 (PMC3142114; doi:10.1371/journal.pone.0021480)

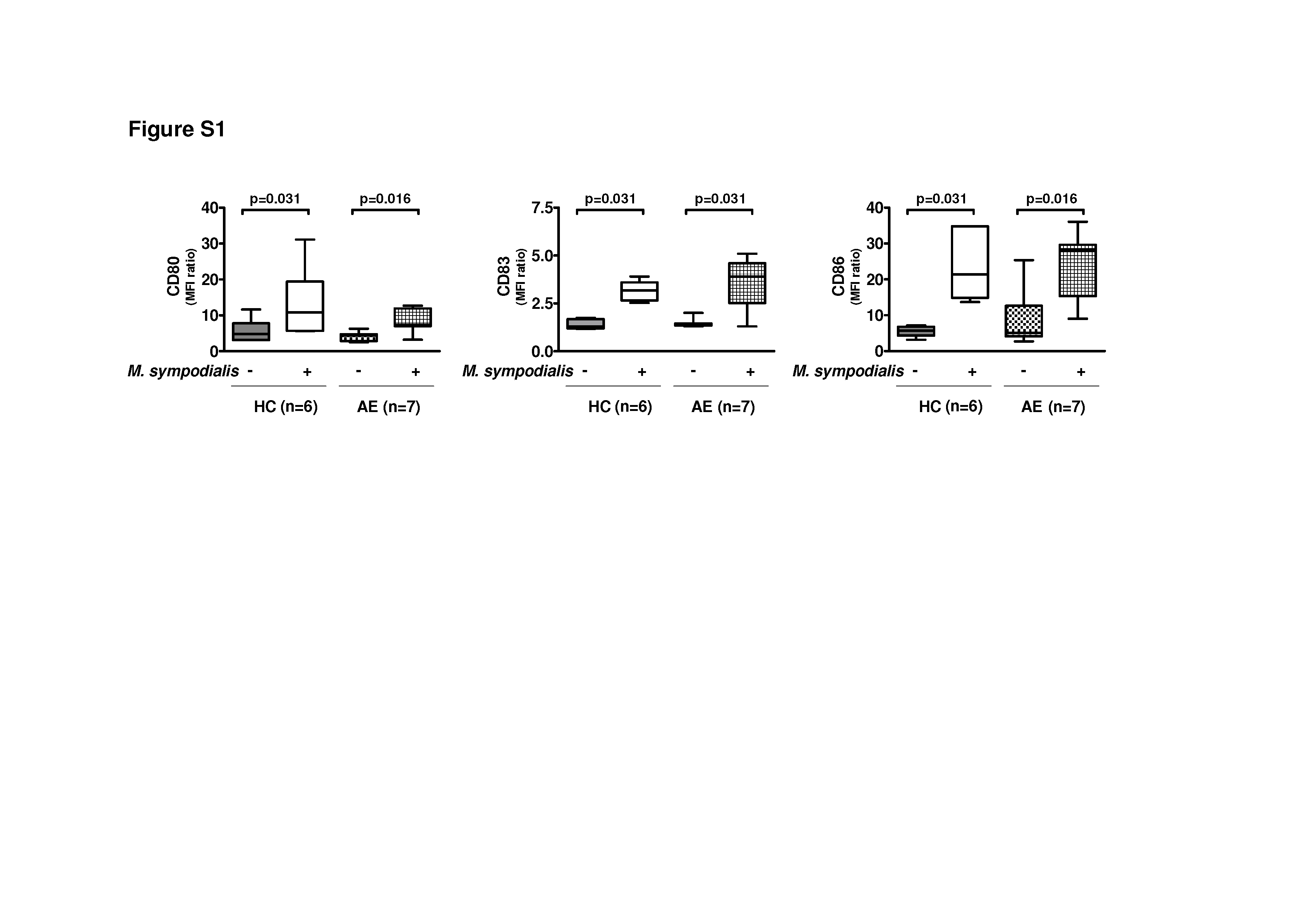

Supplement: Figure S1 — M. sympodialis induces maturation in MDDC. Phenotypes of MDDC generated from AE patients and HC assessed by flow cytometry after 48 hr culture without (−) or with (+) M. sympodialis in a 1∶5 ratio. Data show median of mean fluorescence intensity (MFI) of the sample divided by the isotyope control, 25th–75th percentile and range. (TIF) [file pone.0021480.s001.tif]
