# Supplementary material for: Nanovesicles from Malassezia sympodialis and Host Exosomes Induce Cytokine Responses – Novel Mechanisms for Host-Microbe Interactions in Atopic Eczema
Source: PLoS One. 2011 Jul 22;6(7):e21480. doi: 10.1371/journal.pone.0021480 (PMC3142114; doi:10.1371/journal.pone.0021480)
Supplement: Table S1 — Protein content1) of vesicle preparations. * HC = Healthy control. † AE = Atopic eczema patient. 1)as determined by Bradford assay (BioRad, Hercules, CA). 2)Exosomes were resuspended in RPMI. 3)Exosomes were resuspended in PBS. (PPT) [file pone.0021480.s002.ppt]

## Slide 1
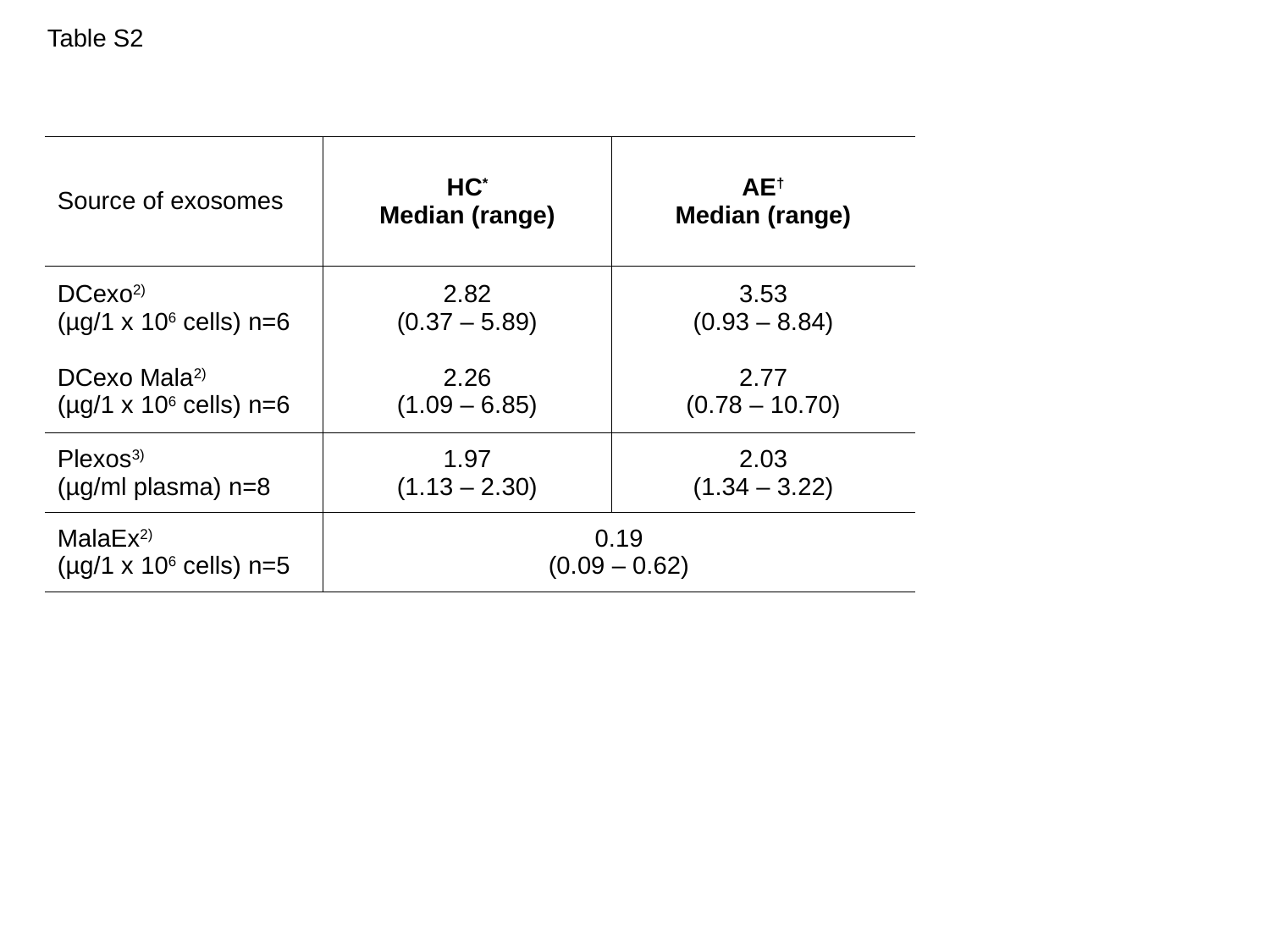

Table S2
| Source of exosomes | HC\* Median (range) | AE† Median (range) |
| --- | --- | --- |
| DCexo2) (µg/1 x 106 cells) n=6 | 2.82 (0.37 – 5.89) | 3.53 (0.93 – 8.84) |
| DCexo Mala2) (µg/1 x 106 cells) n=6 | 2.26 (1.09 – 6.85) | 2.77 (0.78 – 10.70) |
| Plexos3) (µg/ml plasma) n=8 | 1.97 (1.13 – 2.30) | 2.03 (1.34 – 3.22) |
| MalaEx2) (µg/1 x 106 cells) n=5 | 0.19 (0.09 – 0.62) | |
