# Supplementary material for: Nanovesicles from Malassezia sympodialis and Host Exosomes Induce Cytokine Responses – Novel Mechanisms for Host-Microbe Interactions in Atopic Eczema
Source: PLoS One. 2011 Jul 22;6(7):e21480. doi: 10.1371/journal.pone.0021480 (PMC3142114; doi:10.1371/journal.pone.0021480)
Supplement: Table S2 — Exosome-induced cytokine responses in AE patients and healthy controls. § HC = Healthy control. † AE = Atopic eczema patient. ND = not done. 1)as determined by ELISPOT. 2)as determined by ELISA. *Mann-Whitney test to compare AE and HC, p-value<0.05. (PPT) [file pone.0021480.s003.ppt]

## Slide 1
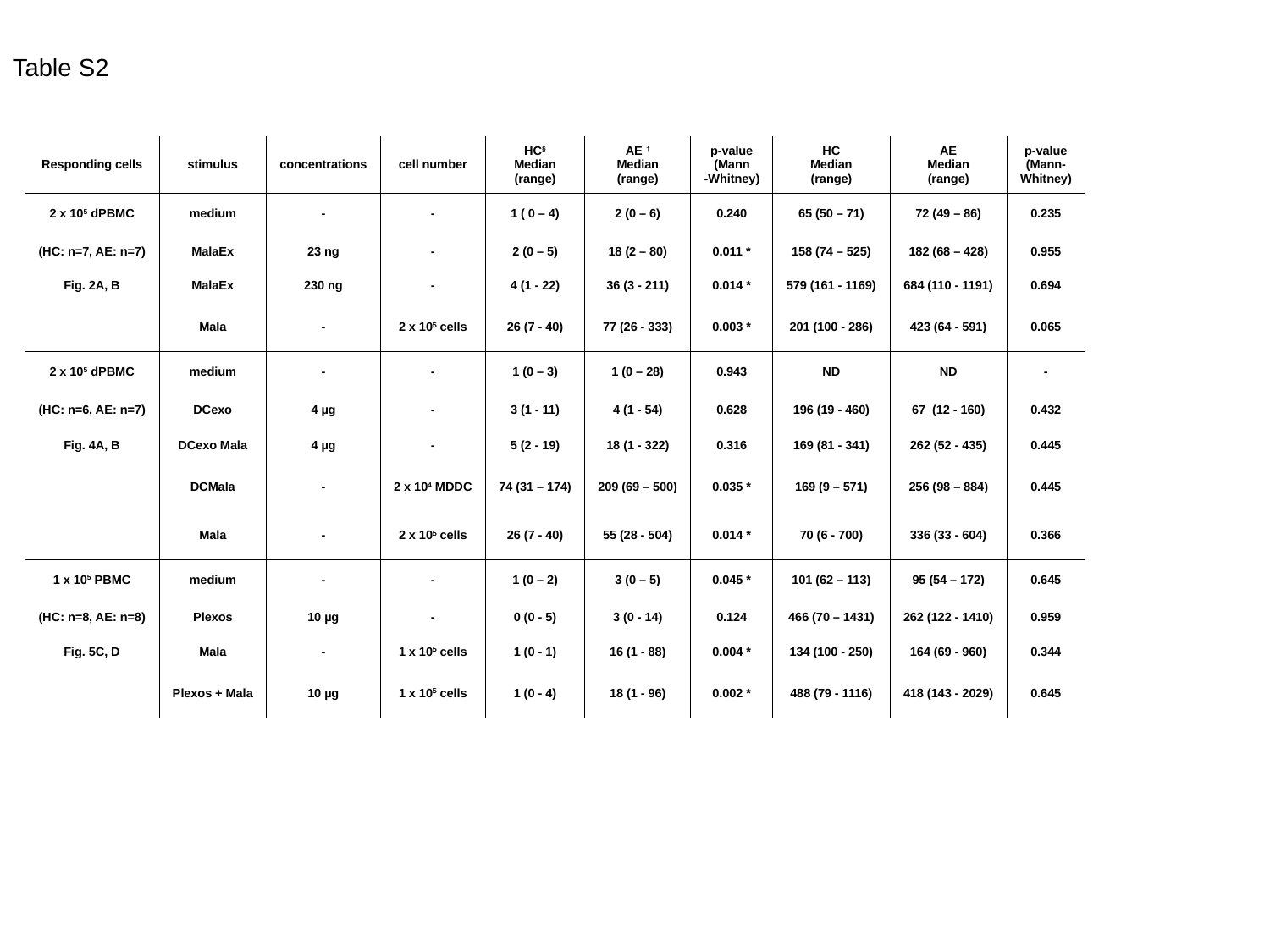

Table S2
| Responding cells | stimulus | concentrations | cell number | HC§ Median (range) | AE † Median (range) | p-value (Mann -Whitney) | HC Median (range) | AE Median (range) | p-value (Mann- Whitney) |
| --- | --- | --- | --- | --- | --- | --- | --- | --- | --- |
| 2 x 10^5^ dPBMC | medium | - | - | 1 ( 0 – 4) | 2 (0 – 6) | 0.240 | 65 (50 – 71) | 72 (49 – 86) | 0.235 |
| (HC: n=7, AE: n=7) | MalaEx | 23 ng | - | 2 (0 – 5) | 18 (2 – 80) | 0.011 \* | 158 (74 – 525) | 182 (68 – 428) | 0.955 |
| Fig. 2A, B | MalaEx | 230 ng | - | 4 (1 - 22) | 36 (3 - 211) | 0.014 \* | 579 (161 - 1169) | 684 (110 - 1191) | 0.694 |
| | Mala | - | 2 x 10^5^ cells | 26 (7 - 40) | 77 (26 - 333) | 0.003 \* | 201 (100 - 286) | 423 (64 - 591) | 0.065 |
| 2 x 10^5^ dPBMC | medium | - | - | 1 (0 – 3) | 1 (0 – 28) | 0.943 | ND | ND | - |
| (HC: n=6, AE: n=7) | DCexo | 4 µg | - | 3 (1 - 11) | 4 (1 - 54) | 0.628 | 196 (19 - 460) | 67 (12 - 160) | 0.432 |
| Fig. 4A, B | DCexo Mala | 4 µg | - | 5 (2 - 19) | 18 (1 - 322) | 0.316 | 169 (81 - 341) | 262 (52 - 435) | 0.445 |
| | DCMala | - | 2 x 10^4^ MDDC | 74 (31 – 174) | 209 (69 – 500) | 0.035 \* | 169 (9 – 571) | 256 (98 – 884) | 0.445 |
| | Mala | - | 2 x 10^5^ cells | 26 (7 - 40) | 55 (28 - 504) | 0.014 \* | 70 (6 - 700) | 336 (33 - 604) | 0.366 |
| 1 x 10^5^ PBMC | medium | - | - | 1 (0 – 2) | 3 (0 – 5) | 0.045 \* | 101 (62 – 113) | 95 (54 – 172) | 0.645 |
| (HC: n=8, AE: n=8) | Plexos | 10 µg | - | 0 (0 - 5) | 3 (0 - 14) | 0.124 | 466 (70 – 1431) | 262 (122 - 1410) | 0.959 |
| Fig. 5C, D | Mala | - | 1 x 10^5^ cells | 1 (0 - 1) | 16 (1 - 88) | 0.004 \* | 134 (100 - 250) | 164 (69 - 960) | 0.344 |
| | Plexos + Mala | 10 µg | 1 x 10^5^ cells | 1 (0 - 4) | 18 (1 - 96) | 0.002 \* | 488 (79 - 1116) | 418 (143 - 2029) | 0.645 |
