# Supplementary material for: Nanovesicles from Malassezia sympodialis and Host Exosomes Induce Cytokine Responses – Novel Mechanisms for Host-Microbe Interactions in Atopic Eczema
Source: PLoS One. 2011 Jul 22;6(7):e21480. doi: 10.1371/journal.pone.0021480 (PMC3142114; doi:10.1371/journal.pone.0021480)
Supplement: Online Repository S1 — (DOC) [file pone.0021480.s004.doc]

**Online Repository**

**Nanovesicles from *Malassezia sympodialis* and host exosomes induce cytokine responses – novel mechanisms for host-microbe interactions in atopic eczema**

**Ulf Gehrmann,^a*^ Khaleda Rahman Qazi,^a^ Catharina Johansson,^a^ Kjell Hultenby,^b^ Maria Karlsson,^c^ Lena Lundeberg,^c^ Susanne Gabrielsson,^a†^ Annika Scheynius,^a†^**

**^a^Clinical Allergy Research Unit, Department of Medicine Solna, Karolinska Institutet, Stockholm, Sweden ^b^Department of Laboratory Medicine, Karolinska University Hospital Huddinge, Huddinge, Sweden and ^c^Dermatology and Venereology Unit, Karolinska University Hospital Solna, Stockholm, Sweden**

**^†^**These authors share senior authorship

^*^Corresponding author:

Ulf Gehrmann

Karolinska Institutet

Department of Medicine Solna

Clinical Allergy Research Unit, L2:04

Karolinska University Hospital Solna

SE-171 76 Stockholm, Sweden

Phone: +46 8 51776696

Fax: +46 8 335724

E-mail: [ulf.gehrmann@ki.se](mailto:ulf.gehrmann@ki.se)

**METHODS**

**AE patients and healthy controls**

Inclusion criteria for the AE patients were diagnosis according to the UK working party [36], moderate to severe eczema and skin lesions not only restricted to the hands. At the first visit, subjects were interviewed about their medical history and the severity of the eczema was assessed using SCORAD [48]. Exclusion criteria were other skin diseases than AE, autoimmune diseases, immune deficiencies and ongoing malignant diseases. Patients were Phadiatop® positive (Phadia AB, Uppsala, Sweden), had specific IgE to *M. sympodialis* (ImmunoCAP^TM^ m70, Phadia AB) and showed a positive atopy patch test (APT) reaction to *M. sympodialis* [32]. Healthy controls had no clinical symptoms or history of allergy or skin diseases and were Phadiatop® negative (Table 1).

**Generation of monocyte derived dendritic cells (MDDC)**

CD34^+^ progenitor cells were removed from the 450 ml blood donation to generate mast cells [37] followed by MACS CD14 positive selection according to the manufacturer’s instructions (Miltenyi Biotech, Bergisch Gladbach, Germany). Purity was assessed by flow cytometry and ranged from 76-96% with a median of 93% (n=15) CD14^+^ cells without any significant differences between HC and AE patients. Viability of CD14^+^ cells was always above 90% using the trypan blue exclusion method. The remaining CD14, CD34-depleted PBMC (dPBMC) were frozen at -150°C in 10% DMSO for later use [38].

**Co-culture of MDDC with *M. sympodialis***

Immature MDDC were seeded at 4 x 10^5^ cells/ml in 175 cm^2^ culture flasks (BDBiosciences, Bedford, MA) and cultured with or without 2 x 10^6^ live *M. sympodialis* cells/ml at 37°C in 6% CO_2_ in complete, exosome-free medium [35]. After 48 hr, cells were pelleted at 300 x g for 10 min and culture supernatants were collected, centrifuged for 30 min at 3.000 x g to remove cell debris and saved at -80°C for exosome preparation. MDDC were phenotyped using flow cytometry (see below) and frozen in 10% DMSO at -80°C for further experiments.

**Flow cytometric analysis of cells**

FITC conjugated mouse monoclonal antibodies (mAb) against the following proteins and their corresponding isotype controls were used to characterize MDDC: HLA-ABC, HLA-DR, CD11c, CD14, CD40, CD54, CD63, CD80, CD83, CD86, (all from BD Biosciences). All antibodies were used according to the manufacturer’s instructions. The analysis was performed on a FACSCalibur (BD Biosciences) using Cellquest software (BD Biosciences). A gate on live cells was set based on forward and side scatter and 1 x 10^4^ gated events were recorded for each sample.

**Nanovesicle preparations**

Exosomes from *M. sympodialis* and MDDC culture supernatants were spun at 10.000 x g for 30 min to remove larger vesicles [34]. The remaining supernatant was pelleted at 100.000 x g for 90 min and washed again at 100.000 x g for 90 min.

Plasma exosomes were prepared similarly as described before with some modifications [8]. Briefly, 200 ml plasma was diluted 1:1 in PBS and spun twice at 11.600 x g for 45 min to remove large protein aggregates. Plexos were then pelleted at 140.000 x g for 2 hr, filtered through 0.22 µm filters (Advantec MFS, Inc., Dublin, CA) and washed twice in PBS at 140.000 x g for 90 min.

**Flow cytometric characterization of exosomes**

DCexo, DCexo Mala and Plexos were coated onto anti-MHC class II coated Dynabeads (custom made with clone HKB1, recognising all MHC class II subtypes; Invitrogen/Dynal, Paisley, UK) over night at room temperature (DCexo and DCexo Mala: 5 µg/µl; Plexos: 15 µg/µl). Beads were washed once and labelled with the following FITC-conjugated mAb to HLA-ABC, HLA-DR, CD63, CD81, CD86 (all BD Biosciences) and PE-labelled mAb to CD3, CD19, CD54 (all BD Biosciences) and the corresponding isotype controls. In addition, Plexos were phenotyped using FITC conjugated mAb against B-cell activating factor (BAFF) (R&D Systems, Minneapolis, MN), FasL (Calbiochem, LaJolla, CA), CD36 (BeckmanCoulter) and PE-labelled mAb against TGFß1 (IQProducts, Groningen, The Netherlands), APRIL (BD Biosciences) and the corresponding isotype controls. Analysis was performed on a FACSCalibur using Cellquest software. A gate was set on single beads and 5 x 10^3^ events were recorded per sample.

**Sucrose gradient analysis**

*M*. *sympodialis*-derived nanovesicles, MDDC-derived exosomes and Plexos were analysed using sucrose gradient fractionation. The two latter were generated from additional healthy blood donors according to the above described protocols, with the exception that the PBMC were not depleted of CD34^+^ cells before culture. For each experiment, exosome preparations (DCexo, DCexo Mala, and Plexos) from two healthy donors were pooled to obtain sufficient material. Nanovesicle preparations were placed on top of a continuous 0.25 M – 2 M sucrose gradient and centrifuged for >14 hr at 79.000 x g [10]. One ml fractions were recovered, density measured using a refractometer (Pleuger, Wijnegem, Belgium) and incubated over night with uncoated 4 µm latex beads (Invitrogen) in PBS or anti-MHC class II Dynabeads (Invitrogen/Dynal) in bead coating buffer (BCB [PBS, 0.5 % BSA (Sigma), 0.01 % sodium acide]). 250 µl of each fraction and 0.25 µl of beads were used for each sample. Dynabeads were washed once in BCB using a magnet while latex beads were incubated in BCB for 30 min, washed in BCB for 15 min at 5.000 x g and incubated with FITC-conjugated mAbs against CD63, CD81 and HLA-DR and their corresponding isotype controls (BD Biosciences) or rabbit IgG raised against *M. sympodialis* extract (generated in house) and normal rabbit IgG (Dako Cytomation). Beads were analysed using flow cytometry as described above. The remaining vesicles from sucrose gradient fractions were pelleted at 200.000 x g for 30 min, resuspended in a small volume of PBS and stored at 4°C.

**ELISPOT analyses**

ELISPOT analyses were performed in 96-well filter-membrane plates coated with 10 µg/ml primary mAb against human IL-4 at 4°C over night. All cultures were run in triplicates and performed at 37°C for 48 hr. dPBMC (2 x 10^5^ cells/well) were cultured alone or with: DCexo (4 µg/well), DCexo Mala (4 µg/well) or MalaExo (23 or 230 ng/well). Co-culture of dPBMC with 2 x 10^5^ *M. sympodialis* cells (Mala) or 2 x 10^4^ autologous MDDC pre-exposed to *M. sympodialis* in a 1:5 ratio (DCMala) served as positive controls. For Plexos, 10 µg were used to stimulate PBMC (1 x 10^5^ cells/well) in the absence or presence of 1 x 10^5^ *M. sympodialis* cells. Plates were analysed using an ELISPOT automated reader (AID Diagnostika, Straßberg, Germany). Results are expressed as the mean number of triplicates of IL-4 spots per 2 x 10^5^ dPBMC or 1 x 10^5^ PBMC for each individual.

**Depletion of MHC class II expressing DC-derived exosomes**

MDDCs were generated from buffy coats from two healthy blood donors and DC-derived exosomes were prepared according to the above described protocols. Ten µg of DCexo and DCexo Mala from both donors were depleted of MHC class II positive vesicles by incubating with 5 µl of anti-MHC class II coated Dynabeads (Invitrogen/Dynal) overnight on a rotator. After incubation, beads with attached vesicles were removed by washing 5 times on a magnet using 0.1% BSA containing PBS. Remaining exosomes were resuspended in complete medium and added at a concentration of 4 µg/well to 2 x 10^5^ autologous PBMC. All cultures were run in duplicates and performed at 37°C for 48 hr. TNF-α levels were measured in the supernatants using ELISA (Mabtech).

**REFERENCES**

36. Williams HC (1995) Atopic eczema. BMJ 311: 1241-1242.

37. Ribbing C, Engblom C, Lappalainen J, Lindstedt K, Kovanen PT, et al. (2010) Mast cells generated from patients with atopic eczema have enhanced levels of granule mediators and an impaired Dectin-1 expression. Allergy no. doi: 10.1111/j.1398-9995.2010.02437.x.

38. Fowke KR, Behnke J, Hanson C, Shea K, Cosentino LM (2000) Apoptosis: a method for evaluating the cryopreservation of whole blood and peripheral blood mononuclear cells. J Immunol Methods 244: 139-144.
